# Societal perspectives on disease and treatment attributes characterizing rare diseases: a qualitative study from the United States

**DOI:** 10.1186/s41687-022-00413-6

**Published:** 2022-01-24

**Authors:** Shelagh M. Szabo, Ivana F. Audhya, David Feeny, Peter Neumann, Daniel C. Malone, Katherine L. Gooch

**Affiliations:** 1 Broadstreet HEOR, Vancouver, BC V6A 1A4 Canada; 2 Sarepta Therapeutics, Inc., Cambridge, MA USA; 3 McMaster University, Hamilton, ON Canada; 4 Tufts School of Medicine, Boston, MA USA; 5 The University of Utah, Salt Lake City, UT USA

**Keywords:** Value of healthcare, Value frameworks, Preferences, Rare diseases, Pediatric diseases

## Abstract

**Purpose:**

Under a societal perspective, disease and treatment attributes that the general public deem important should be considered within value frameworks. The objective was to investigate how members of the general public value attributes beyond health gains and healthcare system expenditures; and better understand their perspectives regarding the importance of attributes typically characterizing rare genetic diseases like Duchenne muscular dystrophy (DMD).

**Methods:**

Qualitative interviews were conducted to elicit feedback on the importance of disease and treatment attributes from general public participants from three US cities. Participants ranked attributes (scale, 1–10) in terms of importance for future research, reported their rationale for ranking, and provided feedback specific to rare diseases. Interview transcripts were coded using NVivo for thematic analysis.

**Results:**

The 33 participants (median age, 51 years; 48.5% male) ranked disease severity (mean [median] ranking, 8.7 [9.0]), treatment availability (8.7 [9.0]), and impact on life expectancy (8.4 [9.0]), as most important. The impact on the family, need for equity, and intrinsic value of life were frequently provided rationales. While rare disease as an attribute received a relatively low ranking (6.1 [7.0]), 88% of participants prioritized disease profiles including attributes of severity, health related quality of life (HRQoL) impact, limited lifespan and young age at onset.

**Conclusion:**

Attributes including disease severity, impact on life expectancy and HRQoL, and treatment availability were all highly important to members of the general public. These findings support the growing evidence regarding the importance of expanding value assessments to include attributes considered important from a societal perspective.

## Introduction

Conventional value assessment frameworks, tools developed by health economic and health professional organizations to guide priorities in healthcare provision, consider elements such as health gains to the patient and costs to the healthcare system when assessing the value of new therapies [1, 2]. The metrics by which these elements are included in cost-effectiveness analyses to estimate the value of new therapies are typically net quality-adjusted life years (QALYs) along with direct and indirect costs. These parameters are used to calculate the incremental costs per QALYs gained with a new health technology relative to the standard of care, and incremental costs per QALY are compared to established benchmarks to assess treatment value [3]. Expanding assessment frameworks that seek to take a more holistic approach to determining treatment value have grown, at least in part, out of a need to inform decision-making for medications for rare and orphan diseases. These frameworks aim to extend beyond the cost per QALY approach by incorporating a broader set of disease and treatment attributes or criteria that presently tend to receive little or no quantifiable consideration under the traditional framework [1].

Such expansions to traditional value assessment frameworks have recently gained attention [1, 4]. In 2018, an International Society for Pharmacoeconomics and Outcomes Research (ISPOR) Special Task Force Report identified and defined novel elements of value that have, to date, been largely unexamined within value assessment frameworks; most of these components would be relevant under a broader societal, rather than narrower health care perspective of valuation [1]. These novel elements included the *insurance value* of new treatments, considering both physical and financial risk protection; the *value of hope,* which considers the impact of individual risk preferences on decision making; *scientific spillover,* where a new technology can benefit future innovation; and the impact of *the severity of disease* [1]. These and other attributes (such as disease rarity, young age at disease onset or the benefit of “buying time”) [4], may be particularly pertinent for life-limiting, progressive and irreversible diseases with a high disease burden and poor prognosis such as Duchenne muscular dystrophy (DMD); diseases for which the impact is felt not by the patient alone, but also their families and potentially other members of society [5].

When a societal perspective is taken into account [6], all disease and treatment attributes that members of society deem important, as well as the impact of interventions on these attributes, should be considered to the extent possible when evaluating the costs and effects of therapies. Therefore, societal views on these attributes must be understood to inform decision making, and better characterize the value of new treatments. However, relatively little data exist on how the general public “values” different disease and treatment attributes [7]. A greater understanding of societal views will help inform whether value frameworks should be modified accordingly. Exploring these views in the context of the attributes of varied health conditions, including life-limiting rare progressive diseases of infancy, may help illustrate some of the complexities to consider when assessing treatment value under a societal perspective [8]. The objective of this study was to investigate how members of the general public value disease and treatment attributes beyond clinical treatment benefits gained by patients and overall costs to the healthcare system; and to ascertain their importance in shaping priorities for healthcare research, from a societal perspective.

## Methods

To understand the importance of disease and treatment attributes from the perspective of members of the US general public, a descriptive qualitative study was conducted [9]. One-on-one in-person qualitative interviews were conducted by three interviewers (SMS, MH and JB) in Seattle, San Francisco, and Dallas. The interview session also included a numeric ranking exercise to assess the importance of disease and treatment attributes relative to one another. The study conduct and reporting were guided by the COnsolidated criteria for REporting Qualitative research (COREQ) [10].

### Identification of disease and treatment attributes

Disease and treatment attributes potentially important for decision-making were identified from several sources, described in more detail below: the publications of the ISPOR Special Task Force on Value Assessment [1, 11]; a targeted literature review; and discussions with a convenience sample of eight members of the US general public. The attributes considered by the ISPOR Special Task Force within any of the five US frameworks that reflected considerations commonly arising in decision-making dilemmas pertaining to rare disease treatments were tabulated [1, 11]. Any additional articles describing value frameworks published since 2018 were identified based on a targeted review undertaken using Pubmed, Google Scholar and an internet search; these publications were also reviewed for further potential attributes relevant to rare diseases [12, 13]. The list of potential attributes was supplemented by review of global health technology assessment agency submission requirements [14–19]. Feedback on the relevance of these attributes for healthcare decision-making was solicited from a convenience sample of eight members of the US general public; these individuals were also asked whether there were any additional attributes they felt would warrant consideration. This convenience sample was recruited from Seattle, Washington in October 2019; and discussions with this sample continued until no new attributes were identified based on feedback from two consecutive participants. The resulting set of attributes was used in discussions during the subsequent qualitative interviews (see below; Qualitative Interview Structure).

To reduce burden in the qualitative interviews, the number of attributes to consider was limited to ten per participant. This number was set based on feasibility feedback from pilot interviews, which were conducted with eight individuals to review the ordering, length, and comprehensibility of the interview materials (see below). As a result, from the full range of potential attributes, the list of attributes for discussion within the qualitative interviews was narrowed down to: disease rarity, age at disease onset, disease cause (genetic vs. acquired), availability of treatments, disease severity, life expectancy, mental health, impact on activities of daily living (ADL) and health-related quality-of-life (HRQoL) and caregiver burden.

The final set of attributes included was selected because it covered a wide breadth of aspects, and were well understood by members of the pilot interview sample (compared to potential attributes such as the *value of hope* or *value of innovation*, for example) [11]. During the subsequent qualitative interviews, participants also had the opportunity to suggest additional attributes they thought important for discussion.

### Qualitative interview participant recruitment

A purposive sample of adults (18 years of age or older) was continuously enrolled for qualitative interviews. Individuals were recruited to generally reflect the age and sex distribution of the US general population; and to include a mix of participants in terms of their familiarity with chronic and rare diseases, and whether they had children living at home. This was undertaken to account for the fact that particular attributes might be predictors of choice in different scenarios. For example, in situations where parents have young children living at home, the *young age at onset* attribute may resonate more acutely. In contrast, for someone with a more intimate experience of chronic diseases, the *rare disease* attribute may hold less weight. Therefore, a recruitment target was set of a minimum 8 participants with familiarity with rare diseases, and 8 participants with children living at home; such that approximately one quarter of the assumed minimum sample size would have experience with these factors, to ensure a diversity of experiences were represented. Level of familiarity with chronic and rare diseases was assessed by asking whether a participant or their family member had, or whether they considered themselves familiar with, any of the items on a list of health conditions that included ‘rare diseases’ as well as other more common conditions (e.g. diabetes, hypertension, dementia).

Consistent with sample size considerations for qualitative studies [20, 21], recruitment of a minimum of 30 participants was planned. Telephone recruitment and screening was carried out by a specialist healthcare market research provider using a panel of potential participants who had been assembled through social media and telephone recruitment. The invitation to participate was provided to panel members from each of the target cities by email, and interested participants directed to contact the recruiters for screening against recruitment criteria. Recruitment continued until information saturation was judged to be reached upon no new themes emerging within three consecutive rounds of interviews.

Prior to initiating data collection, approval from the IntegReview Independent Review Board (IRB) was obtained. Written informed consent was obtained from all participants prior to their interview. All participants were compensated $100 for their time.

### Interview materials

A semi-structured interview guide and set of visualization props were developed. Questions for the interview guide were developed based on literature reviews of US value frameworks and the particular attributes of interest, feedback from a convenience sample of eight members of the general public, and iterative review by the study team. The interview guide consisted of a series of open-ended questions and prompts developed to understand participant impressions of the importance of the attributes of interest; note that participants were not asked to consider the importance of attributes in the context of financial resource restraints (i.e. they were not required to prioritize one attribute at the expense of another). The visualization props included disease-specific infographics created to highlight variability in levels of the attributes of interest, and were used to solicit participant feedback on these. One set of infographics described a series of health conditions that were either: (1) rare with pediatric onset and life-limiting (atypical teratoid/rhabdoid tumor [ATRT]); (2) rare with pediatric onset (inherited retinal dystrophy [IRD], type 1 diabetes [T1DM]); or (3) non-rare and affecting primarily older individuals (type 2 diabetes [T2DM], Alzheimer’s disease [AD]). Health conditions were selected by the study team to reflect varied ages of onsets, severities, types of clinical manifestations experienced, and frequency; these were representative of variability in levels of the attributes of interest. The content of the infographics was developed and validated through feedback from four clinician and two patient experts in disease areas of interest. Note, when participants reviewed these health conditions, they were not anonymized.

While the primary focus of the interviews was to understand individual preferences towards a broad set of disease and treatment attributes relevant to a wide variety of health conditions, during the latter part of the interview, opinions on the importance of attributes specifically characterizing life-limiting rare progressive diseases were sought. A separate infographic was therefore also developed for DMD, which was anonymized as ‘*Disease X*’ within the interview (Appendix Fig. 4); this infographic was discussed at the end of the interview, independently from the other health conditions infographics.

All interview materials were reviewed with the pilot test sample to confirm the ordering, duration, and comprehensibility of the interview materials.

### Qualitative interview structure

Interviews were conducted in-person at dedicated private research interview facilities in Seattle, San Francisco, and Dallas between November 2019 to February 2020 (before the COVID-19 pandemic). These cities were selected to ensure some geographic variability in recruitment; participants from only three cities were included for feasibility. Each interview was conducted in English and lasted approximately 60 min. Interviews were conducted by three interviewers trained in qualitative methods; interviewers practiced together in pilot tests and met regularly to share insights and ensure approaches were standardized as the interviews progressed. No repeat interviews were conducted.

Following review of a brief preamble to introduce the interviewer and the motivation for the research, participants were asked to review the initial set of disease-specific infographics; comment on the attributes (or combinations of attributes) they, a priori*,* viewed as the most meaningful in terms of need for research and treatment; and provide the reason for their responses. Then putting aside the infographics, participants considered each individual attribute, ranking these on a scale of 1 *(not important)* to 10 *(very important)* in terms of their significance for prioritizing research and treatment. Each individual attribute was discussed in depth, with participants reporting on drivers for their choices, and their perceptions of relationships between attributes. Finally, participants ranked the anonymized *Disease X* profile (that described DMD) on a scale of 1 *(not important)* to 10 *(very important)* in terms of priority for research and treatment, and were asked to contrast their perceptions on the importance of research for *Disease X* with those of the attributes reflected within the initial health condition infographics they reviewed. This was to investigate whether in-depth consideration of the attributes might affect overall perceptions of the importance of research for a disease with these specific features. Within this part of the interview, participants also provided feedback on, and a numeric value for, what a ‘rare disease’ meant to them.

At the end of the interview, non-identifying demographic details (age, sex, highest level of education) were collected from all participants. Only the interviewer and the participant were present at the time of the interview; after obtaining participant permission, interviews were recorded and later transcribed. The participants were not contacted in follow-up after the completion of the interview so did not provide their feedback on the transcribed data or the study findings.

### Analysis

Transcripts were reviewed by two of the interviewers (SMS and JB), and the wider study team met regularly to discuss and validate emerging themes and interpretation of the data. Transcripts were independently coded by two study team members (SMS and LP) for analysis.

Thematic analysis was used to explore patterns in responses in accordance with the principles and guidelines described by Braun and Clarke [22, 23]. A theoretical approach to thematic analysis, in which codes were derived from the interview guide a priori and assigned using NVivo, allowed the research team to examine key aspects of the data in-depth. The analysis sought to identify themes regarding: (1) which attributes were important to participants; (2) rationales for attribute importance; and (3) broader reasons for importance that may span across individual attributes. Additional codes were inductively identified throughout the analysis.

Demographic and baseline characteristics of the sample were summarized using medians with ranges and numbers (n) with percentages, as appropriate.

Based on the initial set of disease-specific infographics, participant feedback on the reasons they thought the attributes reflected within the condition profile were important, was tabulated. The latter included reasons such as the impact of the pre-specified attributes, or other self-generated reasons for prioritization.

For each attribute, the mean, median, and range of rankings were calculated across participants, and their rationale for judging why attributes were important was summarized. The frequency of participants reporting interactions between attributes was estimated, as was the percentage classifying an attribute with a rank > 6 (i.e. they ranked an attribute as very [rank 7 or 8] or extremely [rank 9 or 10] important). Mean/median rankings, and the frequency of reporting of interactions between attributes, were summarized visually; relationships between attributes were categorized as moderately linked if 40–60% of the participants described an interaction, and highly linked if > 60% described an interaction. Themes that emerged in discussions of interactions across attributes were plotted and patterns reviewed.

For the *Disease X* (DMD) profile, the mean, median and range of rankings were estimated, the reasons for its importance were compiled, and the percentage of participants ranking the importance of the DMD profile as > 6 was tabulated. How perceptions on attributes reflected within the *Disease X* profile compared with those of the other profiles was summarized. Finally, feedback from participants on what prevalence estimates correspond to a disease being rare were summarized according to the thresholds of < 1:10,000 individuals and < 1:100,000 individuals.

## Results

### Participants

Participants for the qualitative interviews were recruited from Seattle (n = 16), San Francisco (n = 8) and Dallas (n = 9). The final participants were drawn from a pool of 68 interested potential participants. One scheduled participant failed to attend their interview. Recruitment continued until the interviewers determined that saturation had been reached, resulting in a final sample size of 33 participants.

The median (range) age was 51 (26–77) years, 49% were male, 33% lived with children under the age of 18 years, 24% were afflicted or had a family member afflicted by a rare disease, and 30% said they were not personally affected but were familiar with rare diseases. Additional participant demographic characteristics are presented in Table 1.

**Table 1:** Participant characteristics (n = 33)

Characteristic	Overall (n = 33)		San Francisco (n = 8)		Dallas (n = 9)		Seattle (n = 16)	
	n	%	n	%	n	%	n	%
Male sex	16	48.5	4	50.0	5	55.6	7	43.8
Median (range) age (years)	51	(26–77)	52	(30–71)	50	(26–65)	52	(26–77)
Highest education level								
Graduate studies	4	12.1	2	25.0	0	0.0	2	12.5
College/university	20	60.6	6	75.0	7	77.8	7	43.8
Grade or high school	9	27.3	0	0.0	2	22.2	7	43.8
Relationship status								
Single	12	36.4	3	37.5	5	55.6	4	25.0
Married/partnership	19	57.6	4	50.0	4	44.4	11	68.8
Divorced/other	2	6.0	1	12.5	0	0.0	1	6.3
# children < 18 years at home								
0	22	66.7	6	75.0	4	44.4	4	25.0
1	6	18.2	2	25.0	1	11.1	4	25.0
2+	5	15.2	0	0.0	3	33.3	5	31.3
Household income								
Less than 25,000	2	6.1	0	0.0	1	11.1	1	6.3
25,000–49,999	8	24.2	1	12.5	4	44.4	3	18.8
50,000–99,999	9	27.3	0	0.0	3	33.3	6	37.5
100,000–149,999	6	18.2	2	25.0	0	0.0	4	25.0
150,000–199,999	4	12.1	1	12.5	1	11.1	2	12.5
200,000+	4	12.1	4	50.0	0	0.0	0	0.0
Rare disease								
Familiar with a rare disease	10	30.3	2	25.0	1	11.1	7	43.8
Self/family member affected	8	24.2	2	25.0	0	0.0	6	37.5
Not familiar with a rare disease	23	69.7	6	75.0	8	88.9	9	56.2

Participants in the overall sample were recruited according to the broad age and sex distribution of the general public of the US, and to reflect a mix of familiarities with rare diseases

### Ranking of disease profiles

Participants reviewed the initial set of health conditions depicted via infographics and were asked to select the condition that included the attributes (or combinations of attributes) they viewed as most meaningful in terms of need for research and treatment. When providing rationales for their choices, participants cited both the pre-specified attributes as well as self-generated reasons. The three most common reasons highlighted were the high prevalence of the health condition (cited by 52%), the condition being familiar/relatable (40%), and the condition having onset in childhood (by 24%).

### Attribute rankings

**Fig. 1:**
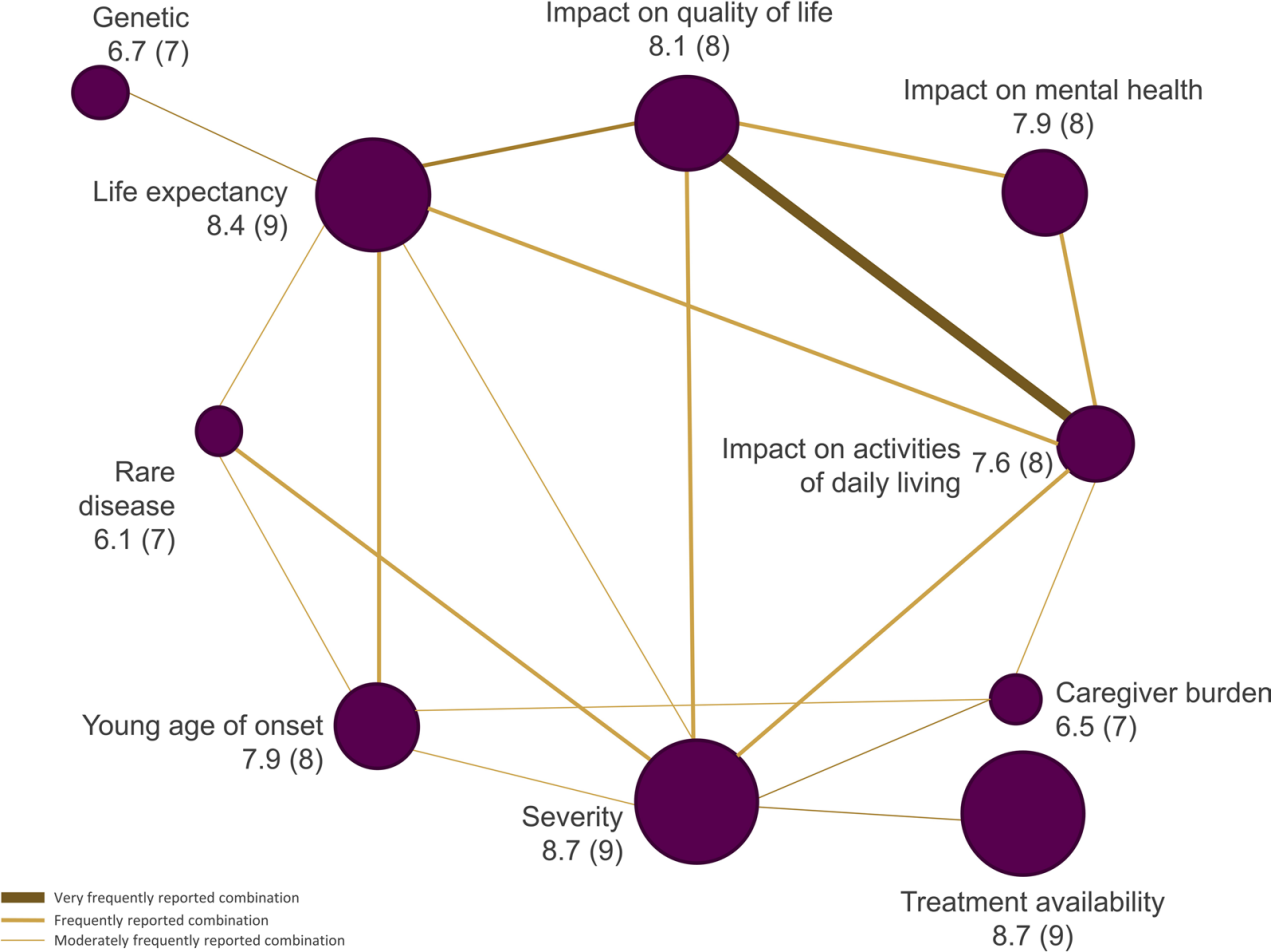
Mean (median) attribute ranking, and frequency of reported relationships between attributes

**Fig. 2:**
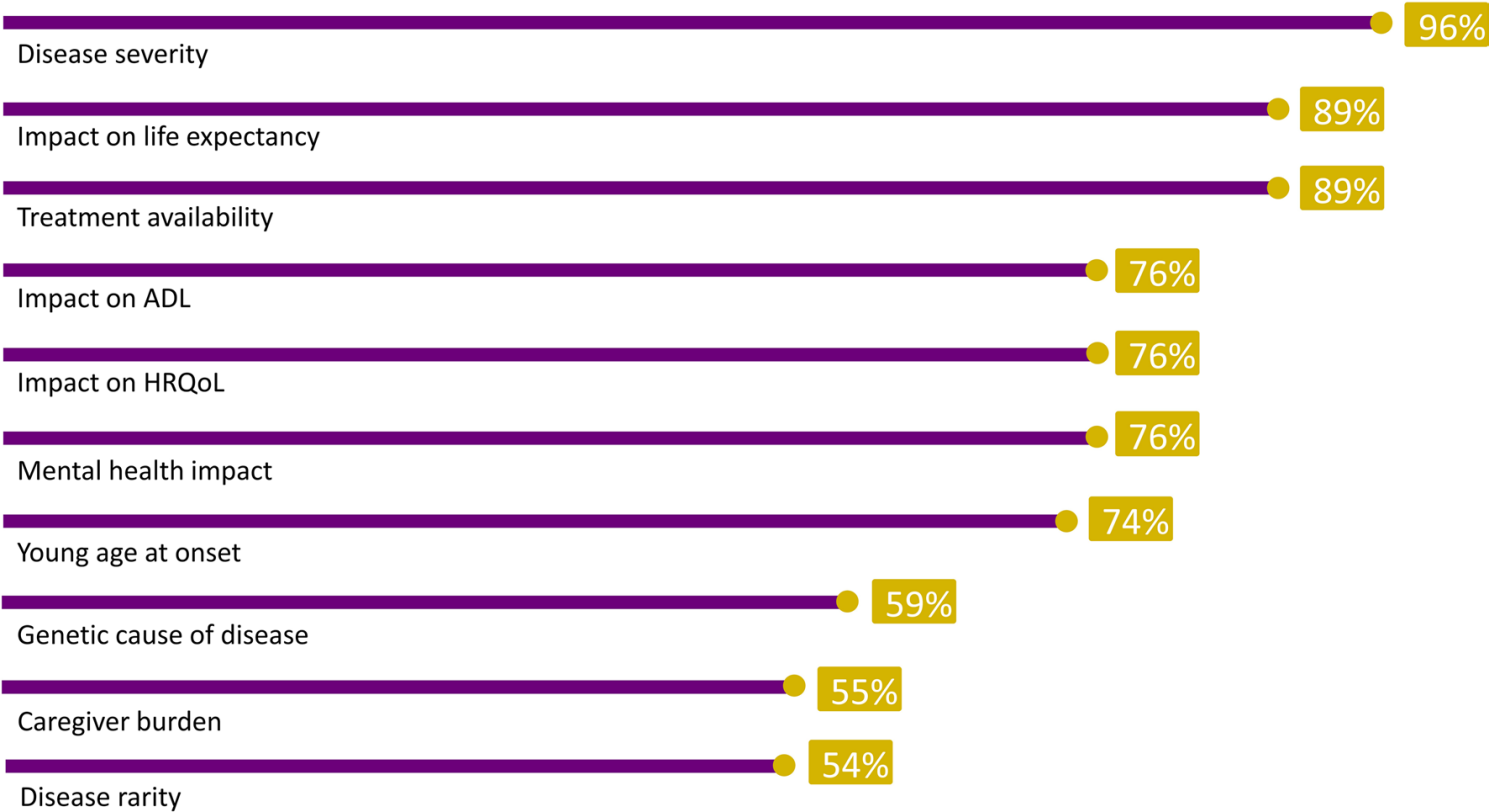
The frequency with which participants ranked attributes as *very* or *extremely important* (n = 33)

**Fig. 3:**
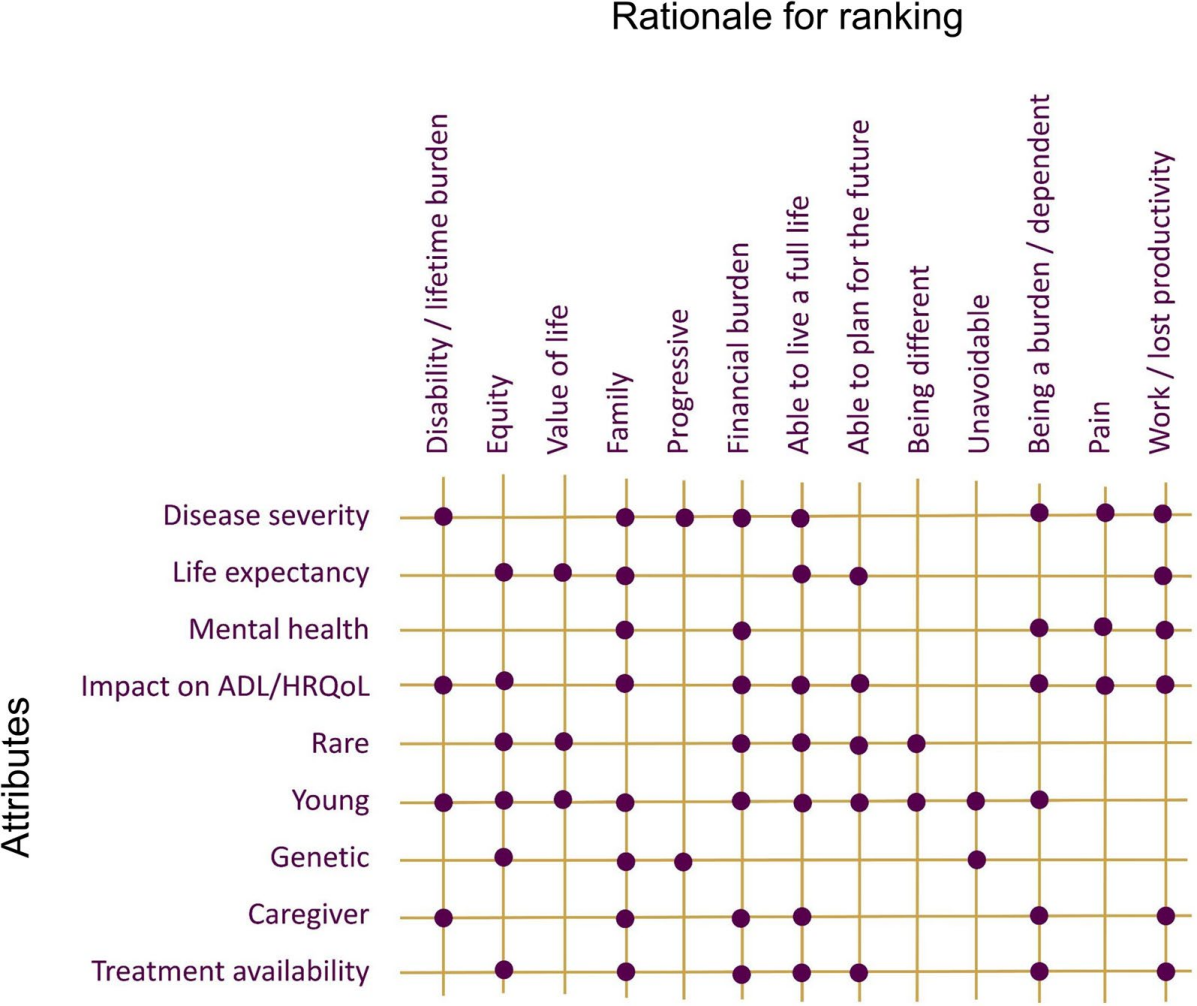
Patterns in rationales for ranking different attributes important for research and treatment

Across all disease and treatment attributes considered, mean (range) rankings ranged from 8.7 (5–10) for disease severity (median, 9.0), to 6.1 (2–10) for rare disease (median 7.0; Fig. 1). Participants provided additional detail on why these highly ranked attributes were important to them (see Table 2 for relevant quotations). Estimates of how frequently individuals ranked individual attributes as *very* or *extremely important* ranged from 96% (disease severity) to 54% (disease rarity; Fig. 2). Varied rationales were provided by participants as to why attributes resonated. These included avoiding disability or lifetime burden, pursuit of equity, the intrinsic value of life, the ability for one to live a full life and plan for the future, impact on the family and to avoid being a burden (Fig. 3). For those who did not highly value caregiver burden, participants noted that while alleviating caregiver impact was important, focusing on treatment of the patient’s underlying disease would be the optimal strategy to mitigate this concern. In general, participants did not distinguish between attributes that would be important for guiding research as compared to those for guiding treatment priorities.

**Table 2:** Participant rationales for the perceived importance of highly ranked attributes

Themes (mean ranking)	Sample quotes
Disease severity (8.7)	*I think that research [into severe disease is] more important because…you really want to try to help people who are suffering…live their life as best as they can*
	*[A severe disease] impacts more than just the person who has it. – Participant 10 (San Francisco)*
Treatment availability (8.4)	*If there's no treatment available, it makes me wonder why isn't anyone doing any research to find out at least something that may help alleviate some of the suffering that a person's going through – Participant 22 (Seattle)*
	*If you have a medication that can help, then it’s highly important that patients who need it get to have it, and [we] need to figure out a way that they can afford it and have access to it… And not too many red tapes (sic) to get it. – Participant 6 (Dallas)*
	*That’s the one that hit me, where I don’t think they got a fair share. For the people that are passing because of lack of treatment, I think…it was because we should have done better as a society trying to find treatment for it. It’s not their fault that people got greedy. It wasn’t their fault that we can’t help them…if we can find a way to do it fairly. Participant 28 (Seattle)*
Impact on life expectancy (8.4)	*You want to be able to save a life, especially a young life – Participant 15 (San Francisco)*
	*It’s really not fair. It’s not fair to the parents who have to suffer through watching someone they brought into the word decline. It’s not fair to the person who is declining…Everyone should ideally have a life expectancy that is pretty equal. Participant 10 (San Francisco)*
Impact on HRQoL (8.1)	*I would be miserable if I couldn’t do the things I normally do…I don’t know that I’d want to live many years depending on someone else to have to cart me around and take care of me*.- *Participant 5 (Dallas)*
	*When people, or especially when kids, can’t do certain things…or participate in certain activities, that’s not fair…people should all have the same opportunities to participate in life…and if something can be done about it, maybe they could push for the research into it? – Participant 20 (Seattle)*

See Fig. 1 for a complete list of attributes and their rankings

Some attributes were frequently discussed in combination with participants noting interactions between them (indicated by the weight of the line in Fig. 1). HRQoL and ADL were discussed in an apparently interchangeable fashion by participants, despite these constructs receiving different importance scores in the ranking exercise. Other attributes frequently reported to occur in combination included: impact on life expectancy (with impact on ADL/HRQoL, genetic cause of disease, and young age at onset) and disease severity (with impact on ADL and caregiver burden).

### *Disease X* profile

The mean (range) ranking assigned to the *Disease X* profile (which was developed to represent DMD), in terms of its importance for research and treatment, was 8.6 (2–10; median, 9). The vast majority (88%) of participants rated the *Disease X* profile as *very* or *extremely* important for research and treatment, and the attributes participants most frequently identified as dictating the importance of this profile were disease severity/impact on HRQoL and ADL (83%), the limited lifespan (57%) and young age at onset (50%; Table 3). Rationales provided for the *Disease X* profile ranking included consideration for equity, the large burden, family impact, unavoidability, value of life, and timing of loss of life. Participants also cited that it was the collection of individual attributes (e.g. the combination of being a life limiting disease, with being a severe disease, with having pediatric onset) that made the *Disease X* profile very important to them.

**Table 3:** Attributes identified by respondents as important for DMD research and treatment (n = 30)

	n	%
Severity/impact on ADL/HRQoL	25	83.3
Limited lifespan	17	56.7
Young age at onset	15	50.0
Inherited/genetic/occurs in families	5	16.7
Lack of treatment availability/no cure presently	4	13.3
Impact on family/caregivers as well as patient	4	13.3
Rarity/disease prevalence	3	10.0

Attributes reported by a minimum of 10% of participants. Participants could specify more than one attribute as being important for research and treatment. Note only 30 participants responded to this set of questions, due to time constraints during the interview

The high ranking assigned to the *Disease X* profile contrasts with the relatively low ranking assigned to the “rare” attribute in the attribute rankings exercise. The mean ranking of the *Disease X* profile was 8.6, corresponding to a status of being *extremely important for research and treatment.* However, the attribute ‘rare’ achieved the lowest mean ranking of all the attributes, at 6.1. This contrast may have occurred because most participants did not think diseases occurring as frequently as DMD (affecting an estimated 1:5000 live male births) [24] were, in fact, ‘rare’. Specifically, 89.5% noted that they thought a rare disease would affect < 1:10,000 (i.e. ~ 33,000 Americans), and 78.9% thought a rare disease would affect < 1:100,000 (i.e. ~ 3300 Americans).

## Discussion

To date limited studies have been conducted to elicit the views of the general public on the importance of disease and treatment attributes beyond health gained by patients and overall costs to the healthcare system. In this study, attributes including disease severity, the impact on life expectancy and HRQoL, a young age at onset, and treatment availability were all highly ranked by members of the general public in terms of their importance for guiding research and treatment into diseases with these attributes. While some of these attributes are considered within traditional value assessment frameworks—notably, impact on life expectancy and HRQoL—others, such as young age of onset or treatment availability, are not considered directly [1, 4]. Some of the attributes most highly ranked in this study were consistent with suggested expansions to existing value frameworks, such as consideration of disease severity and equity [1]. Participants in the current study cited examples of equity that included ensuring that if a treatment is available, everyone eligible can access it; or if there is no treatment available for a given disease, that research into potential treatments become a focus [1]. In addition to these attributes, members of the general public also called out the importance of some other considerations (for example, diseases with a substantial impact on the family or caregiver burden). Some of the attributes prioritized by members of the general public identified in this study, largely ‘sit outside’ current value frameworks and value assessments.

A strength of this study was its ability to provide insight into why members of the general public found specific disease and treatment attributes compelling and numerous explanations were provided—including the burden a disease placed on the patient or family, the value of life, and need for equity. Nonetheless, comparing rationales for “importance rankings” across attributes revealed that, in general, the attributes considered most important, were underpinned by the same rationale. As an example, the impact on family was raised as a key consideration for all of the most highly-ranked attributes. This study also provided insight into how members of the general public viewed the cumulative impact of different levels of, or combinations of, disease and treatment attributes. The rationales provided for which health condition profile reflected attributes meaningful for research and treatment, also revealed that participants went beyond the initial core list of attributes to highlight other self-generated features that resonated with them.

Participants in this study did not seem to value “rarity” independently from other factors, nor necessarily support prioritization of research and treatment for rare diseases over common diseases. Nonetheless, they ranked the DMD profile highly in terms of importance for research and treatment. There are a number of possible explanations. First, participants had differing perceptions as to what the prevalence of a disease would be, to be considered “rare”. This suggests that societal perspectives on the definition of disease rarity may differ from those used by healthcare decision makers, definitions which themselves vary by jurisdiction [25–27] and reflect the lack of consistency in views of members of the general public as to what constitutes a rare disease. Second, it may be the collective impact of the attributes that that drive perceptions of importance rather than the contribution of individual attributes alone. Several other studies have examined the impact of rare diseases on preferences for priority setting. While a number of these reported that rare diseases were not a particular focus for prioritization [28–32], others noted that, all else being equal, members of the general public would support giving priority to a smaller but more severely ill group of individuals, if the disease severity was sufficiently great [33]. Considered together, these findings reinforce the notion that in isolation, disease rarity may not be as compelling as when considered in combination with other disease and treatment attributes.

Many participants focused on the importance of factors such as equity, life expectancy, and young age at disease onset, in defining which diseases should be the focus for research and treatment. This is consistent with the idea of *fair innings*—that everyone should have an equal chance at an equal lifespan [34], which has also previously been cited as a consideration for funding rare disease research and treatments [4]. These findings lend support to the idea that young age at disease onset is an important attribute to consider, from a societal perspective, in the value frameworks..

The considerations identified by participants on the importance of disease and treatment attributes in the present study, have also been identified as relevant themes in other studies taking a societal perspective but with slightly different decision problems. Overall, the evidence on this topic is limited. A survey of 294 member of the general public in ten European countries examined views on healthcare priority setting highlighted five key viewpoints, many of which overlap with the attributes and rationales identified in the current study. These included (1) equity and equality of access; (2) severity and magnitude of health gains; (3) fair innings; (4) the intrinsic value of life; and (5) that quality of life is more important than simply staying alive [7]. The rankings for the attributes estimated in the present study are also in line with the rankings from a Korean study of general population preferences that aimed to understand what should inform reimbursement decisions in oncology from a public payer perspective [35]. That study looked at eight criteria, a mix of those typically considered and those not, including disease severity, treatment efficacy, cost-effectiveness and budget impact, age at onset [‘pediatrics targets’], rarity [‘population size’], innovation and treatment availability [‘unmet need’]. In that study, treatment efficacy, cost-effectiveness, and disease severity were ranked the most important considerations for reimbursement decision making from the general public perspective [35]. What is consistent across studies, is that the members of the general public included in these studies consider a wider array of attributes to have bearing on healthcare focus, than those considered in traditional value frameworks.

While identifying which attributes warrant inclusion in expanded value frameworks is important, the exact mechanisms by which attributes should be incorporated is likely to vary by context. While the mechanisms for including some elements may be relatively straightforward—e.g., aspects of caregiver burden could be considered via caregiver utility values or monetized in the numerator of a cost-effectiveness ratio, for example [36]—others represent more of a challenge. Nonetheless, the methods for considering aspects not presently included in existing value frameworks are evolving [14, 37–39]. Salomon et al., comprehensively summarize potential factors to be considered in cost-effectiveness evaluation under differing perspectives, providing worked examples in the context of alcohol use disorders and end-of-life care, Reed et al., recently employed a discrete choice experiment framework to quantify the value of hope from the patient perspective [38]. With respect to disease rarity, the National Institute of Health and Clinical Excellence (NICE) in England and Wales has modified their evaluation framework for medications for ultra rare, severe and debilitating conditions by increasing the cost-effectiveness threshold to £100,000 per QALY [14]. However, the exact rationale underlying the decision—whether it was due to societal impressions of the importance of rarity, or whether NICE decisionmakers were acting based on their own perceptions of importance of this and other potentially related attributes like young age at onset—has not been reported. What is clear is that incorporating these additional attributes would require varied and flexible valuation methods that synthesize data on these aspects that are currently ‘silent’ in formal cost-effectiveness evaluations, but represent factors also considered important to patients, caregivers, and society.

Strengths of our study included the relatively large sample size for qualitative interviews [20, 21, 40–42]; the consistency in responses given by participants which provides evidence that participants understood exercise and information saturation was achieved; and the alignment between what study participants self-generated as important, with the *ex ante* considerations of the study team in initial outlining these potentially important attributes. Limitations include that, although the sample size was large for a qualitative study, it was not sufficiently large to identify how key factors such as geography or other potential predictors of preferences might affect results. Data on race and/or ethnicity were not collected which is another limitation. As such, this represents an avenue for further research, and conducting a larger quantitative preference-based study would also allow the recruitment of a sample for which the findings would more likely generalize to the US population. Another limitation relates to questions about attribute importance being framed in the context of ‘importance for research or treatment’ and it should be acknowledged that these are two different constructs; individual priorities could have differed if these constructs were considered separately. Nonetheless, participants did not noticeably distinguish these within the discussions of each attribute. The interviews were designed to solicit participant feedback on a wide set of disease and treatment attributes, before finally focusing on the disease profile describing DMD. While DMD was selected as a case study for understanding preferences related to rare pediatric diseases, findings from this section of the interviews may not be broadly generalizable to other health conditions with features that differ markedly from those associated with DMD. Finally, it should be noted that the interviews were conducted just prior to the COVID-19 pandemic and it is possible that participants’ views may now differ.

## Conclusions

Findings from this study highlight disease and treatment attributes valued by this sample of members of the general public, that may help inform future research priorities and strategies seeking to incorporate societal perspective into value frameworks. In addition to attributes accounted for within existing value frameworks such as impact on life expectancy and HRQoL, attributes such as disease severity, treatment availability and unmet need, the age of disease onset, and impact on the wider family unit were also important from a societal perspective; these currently have limited visibility and typically minimal contributions within conventional value assessments. Further research should quantify preferences for these attributes among a large representative sample of members of the general public; and explore methodologies for expanding value frameworks to include disease attributes that are important from societal perspectives.

## Figures and Tables

**Fig. 4:**
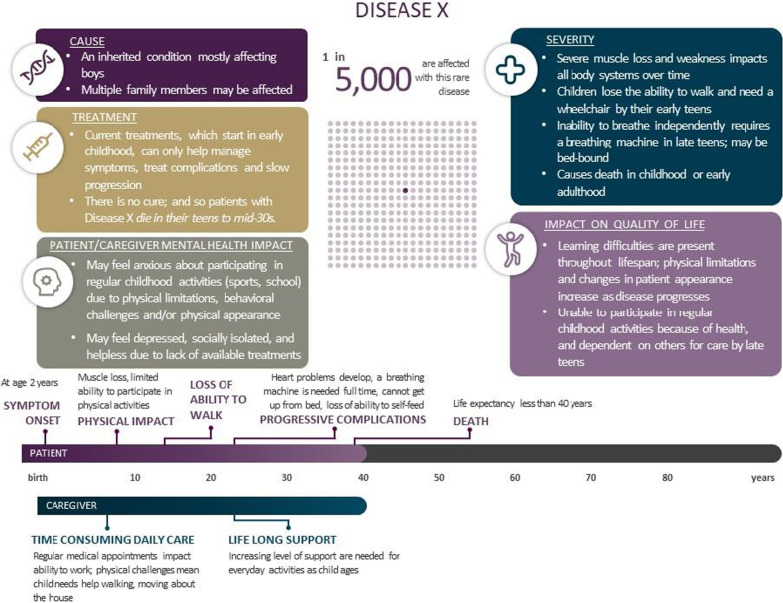
Infographic developed to describe *Disease X*

## Data Availability

Further details of the data are available from the authors upon request.

## Appendix

See Fig. 4.

## Abbreviations

ADL: Activities of daily living
ATRT: Atypical teratoid/rhabdoid tumor
DMD: Duchenne muscular dystrophy
HRQoL: Health-related quality-of-life
IRB: Independent Review Board
ISPOR: International Society for Pharmacoeconomics and Outcomes Research
QALYs: Quality-adjusted life years
SDs: Standard deviations

## Code availability

NA.
